# Association Between Trunk Fat Mass Index and Diabetes in a Multinational Population

**DOI:** 10.1016/j.mayocpiqo.2025.100658

**Published:** 2025-09-19

**Authors:** Chibueze Ogbonnaya, Madison Kindred, Carl J. Lavie, Hannah Oh, Min-Jeong Shin, Xuemei Sui, Jason Jaggers, Ryan Porter, Dahyun Park, Jin E. Kim, Jessica Gong, Vivek K. Prasad

**Affiliations:** a Population, Policy and Practice Research and Teaching Department, University College London, London, United Kingdom; b Department of Kinesiology, Augusta University, Augusta, Georgia; c Department of Cardiovascular Diseases, University of Queensland School of Medicine, New Orleans, Los Angeles; d Department of Public Health Sciences, Korea University, Seongbuk District, Seoul, South Korea; e Department of Exercise Science, University of South Carolina, Columbia, South Carolina; f Department of Health & Sport Sciences, University of Louisville, Louisville, Kentucky; g Department of Kinesiology, Harris College of Nursing & Health Sciences, Texas Christian University, Fort Worth, Texas; h Department of Epidemiology & Public Health, University College London, London, United Kingdom; i Division of Surgery & Interventional Science, University College London, London, United Kingdom

## Abstract

**Objective:**

To examine the cross-sectional association between trunk fat mass index (TFMI) and diabetes across individuals within the same body mass index (BMI [calculated as the weight in kilograms divided by the height in meters squared]) categories in a multinational population.

**Participants and Methods:**

We harmonized and pooled data on 57,764 individuals aged 40 years and older from the United Kingdom, the United States, and South Korea. Trunk fat mass imaging was performed using a dual-energy X-ray absorptiometry device during 2015-2023 in the United Kingdom, 2011-2018 in the United States, and 2008-2011 in South Korea. The prevalence of diabetes was derived from the self-reported medical history. Additionally, plasma biochemistry analyses were conducted to update the number of participants with diabetes.

**Results:**

Among participants classified as having a normal weight based on BMI, the relative risks (RRs) of diabetes increased from TFMI quintiles 1 to 5 with the linear trend (*P*<.001). The risk of diabetes among individuals in TFMI quintile 5 was around 3 times greater than those in quintile 1 (men—RR, 3.06; 95% confidence interval [CI], 2.17-4.34; women—3.35; 95% CI, 2.08-5.39). This significant linear trend (P<.001) in RRs was also present in overweight and obese individuals (overweight men—RR, 1.92; 95% CI, 1.50-2.47; overweight women—RR, 2.25; 95% CI, 1.73-2.91; obese men—RR, 2.47; 95% CI, 1.83-3.35; obese women—2.79; 95% CI, 2.04-3.83).

**Conclusion:**

Within a specific BMI category, individuals with a high trunk fat mass are more likely to experience diabetes compared with those with lower levels of central fat.

Body mass index (BMI [calculated as the weight in kilograms divided by the height in meters squared]) is used worldwide to classify obesity, assuming it is a measure of unhealthy body composition (BC), which is defined as having high body fat percentage (BF%), lower than the required essential body fat (BF), low muscle mass, or high abdominal fat accumulation.^1^, ^2^, ^3^, ^4^ However, unhealthy BC is not confined to the obese, overweight, and underweight BMI categories; it is also prevalent in the normal-weight population.^1^^,^^5^ Therefore, key parameters that should be considered when assessing obesity are BF% and adiposity distribution.

Metabolism in skeletal muscle, adipose tissue, and liver plays an important role in regulating blood glucose homeostasis. Muscle mass accounts for the storage and metabolism of around three-fourths of the postprandially produced glucose and is considered the primary organ accountable for whole-body glycemic control.^6^^,^^7^ On the contrary, adipose tissue is a vital organ that modulates metabolism to meet the body’s varying nutritional demands.^7^ Moreover, adipose tissue functions as an endocrine organ by releasing hormones that influence insulin sensitivity and glucose regulation.^7^ However, excess fatty tissue is detrimental to glucose metabolism, increasing the risk of developing type 2 diabetes mellitus (T2DM).^7^^,^^8^ Similarly, fat distribution parameters, including visceral adipose tissue and ectopic fat on the organs and skeletal muscle, are the critical determinants of insulin sensitivity.^9^, ^10^, ^11^, ^12^

There is strong evidence that individuals with a higher BMI are at increased risk for diabetes.^13^, ^14^, ^15^, ^16^, ^17^ However, on the contrary, a number of studies exhibited that individuals who are overweight or obese with a diabetes history have better prognoses and low mortality rates than the normal-weight counterparts.^18^, ^19^, ^20^, ^21^ One possible theory that could explain the disparities in the research findings is varying levels of abdominal fat or differences in body adiposity distribution across individuals with the same BMI. Additionally, excess central fat deposits among those classified as having a normal BMI may remain an unrecognized risk factor before the diagnosis as well as during the course of diabetes.^22^^,^^23^ Therefore, it is important to investigate the risk of diabetes across individuals with different levels of central fat but categorized within the same BMI range. Our study estimated the risk of diabetes across quintiles of trunk fat mass index (TFMI) within various BMI groups. We hypothesize that high levels of trunk fat mass (TFM) are associated with a greater risk of diabetes, regardless of whether the individuals are classified as normal weight, overweight, or obese based on their BMI. Additionally, the relationship between TFMI and diabetes was examined in the United Kingdom, United States, and South Korean populations to determine whether the association follows a similar pattern in all countries.

## Participants and Methods

Data were received through the BC Studies Collaboration initiative, facilitating building a global consortium of researchers studying body fatness measured by an imaging method like dual-energy X-ray absorptiometry (DXA). For this analysis, we pooled data from the UK Biobank (UKB) study, the US National Health and Nutrition Examination Survey (NHANES), and South Korea NHANES (KNHANES) to create a central data set. Detailed methodologies and standard procedures for data collection in the study and surveys could be found on the respective websites and in data resource articles.^24^, ^25^, ^26^, ^27^ The US and Korean NHANES data sets are publicly available, whereas UKB data were accessed through a formal request submitted to Oxford University. The KNHANES data, originally in Korean, were translated into English by our collaborators at Korea University. The US NHANES and KNHANES are nationally representative health examination surveys, and the UKB is a multicountry population-based project encompassing England, Scotland, and Wales. To investigate the association between diabetes and TFMI, we harmonized data from 57,764 individuals. Participants in the UKB were aged 40 to 83 years, and those in the KNHANES were aged between 40 and 80 years. The US NHANES included individuals aged 40 to 60 years. The UKB study received approval from the North West Multi-centre Research Ethics Committee. The US NHANES approval was granted by the National Center for Health Statistics Research Ethics Review Board, and the Institutional Review Board of the Korea Disease Control and Prevention Agency provided approval for the KNHANES. All participants in the United Kingdom, the United States, and South Korea signed the informed consent forms.

### Dual-Energy X-ray Absorptiometry Assessment and Diabetes Prevalence

In the US and South Korea NHANES, the TFM scans were performed during 2011-2018 and 2008 -2011, respectively, using a Hologic QDR 4500A fan-beam densitometer. The GE-Lunar iDXA device was used to measure TFM in the UKB study from 2015 to 2023. Trunk fat mass is the fat tissue that is located between a horizontal line drawn through the lower end of the chin and the lower border formed by the oblique lines passing through the hip joints. Trunk fat mass index was calculated by dividing TFM in kilograms by height in square meters.

Diabetes prevalence was estimated from a self-reported medical history data, which included diagnoses made by a doctor or the use of diabetes medications. Furthermore, plasma biochemistry analyses were performed to determine HbA1c levels in the UKB study and the US NHANES. Fasting blood glucose levels were measured in the South Korea NHANES. In the UKB study and US NHANES, an individual was considered to have diabetes when the HbA1c level was 6.5% or more. Participants with fasting plasma glucose levels of 126 mg/dL and more in the South Korea NHANES were considered to have diabetes. For the UKB study, participants completed a touch screen questionnaire, followed by an interview that included a comprehensive disease and medication history of diabetes. In the US and South Korea NHANES, diabetes status and drug history were collected through in-person interviews.

### Covariates

Age and sex information in the UKB study was securely obtained from the National Health Services and updated by participants during the verbal interviews. In the US and South Korean NHANES, age and sex were self-reported during personal interviews. Smoking status was self-reported, and participants were characterized as never smokers, past smokers, or current smokers. Body mass index was calculated using weight and height, which were measured during physical examinations. Participants self-reported ethnicities in the UKB study and the US NHANES, whereas KNHANES exclusively included Koreans by ethnicity.

### Data Harmonization

To ensure the validity of scientific output, the central data set was developed to achieve uniformity by harmonization of the variables while accepting a certain level of heterogeneity across the data from the United Kingdom, United States, and South Korea. The approaches that were used for harmonizing the variables include standardization methods (aligning with international classifications and standards), calibration (conversion of units), algorithmic transformation (recoding), and direct mapping (ensuring the target variable is similar to the original variable).^28^^,^^29^ The harmonization table (Supplemental Table 1, available online at http://www.mcpiqojournal.org) underwent multiple rounds of review by team members through teleconferences and electronic communication. Information on missing metadata in the data sets was sought through regular queries to the collaborators.

### Statistical Analyses

Participants were categorized into quintiles based on the TFMI (quintile 1 included individuals with the lowest TFMI, and quintile 5 comprised those with the highest TFMI). Descriptive analysis summarized numeric variables; means with SDs were reported for the normally distributed data, and medians with interquartile ranges were presented for nonnormally distributed data. Categorical variables were summarized by reporting the frequency and percentage of the observations within each quintile.

A modified Poisson regression model was fitted to investigate the cross-sectional relationship between TFMI and diabetes, while accounting for the confounding factors, including age, gender, ethnicity, smoking status, and BMI. We reported regression coefficients as relative risks (RRs) with 95% confidence intervals (CIs) and *P* values for statistical significance. A sensitivity analysis was conducted to compare the RRs from the modified Poisson model with the odds ratios (ORs) from the logistic regression model. It is known that the results presented as RRs may be more intuitive to interpret than ORs. Predictor variables, including sex and age, were added to the regression model due to clinical relevance. Other variables were added when they improved the model fit as evaluated by the Bayesian information criteria. Regression models with lower Bayesian information criterion values were preferred in order to attain a balance between complexity and fit.

An elaborated subgroup analysis was conducted to investigate the relationship between TFMI and diabetes status among men and women within different BMI categories and nations. The BMI groups were categorized as underweight (<18.5 kg/m^2^), normal weight (18.5-24.9 kg/m^2^), overweight (25-29.9 kg/m^2^), and obese (≥ 30 kg/m^2^) for populations in the United Kingdom and the United States. For the South Korean population, BMI was defined according to the classification system used in Korea as underweight (<17.5 kg/m^2^), normal weight (17.5-22.9 kg/m^2^), overweight (23-27.9 kg/m^2^), and obese (≥28 kg/m^2^). Line graphs for differences in mean TFMI between individuals with diabetes and those without diabetes were constructed across the BMI categories.

Of 57,764, 958 (1.66%) participants in the central data set had at least 1 missing value. In the UK, US, and South Korea cohorts, the number of individuals with incomplete data was 486, 348, and 124, respectively. The variables with the highest proportions of missing values were smoking (0.80%) and TFM (0.63%). On investigating the missing data mechanism, it was assumed that the unavailable values are missing completely at random. This indicates that the observed information in the analysis and the probability of data being unavailable are unrelated. Listwise deletion was used to address the missing data, a technique in which the participants with at least 1 missing value are excluded from the regression analysis. We conducted a sensitivity analysis to explore the impact of the missing data by comparing the results of the analysis when the listwise deletion method was used vs when the multiple imputation procedure (a technique that uses statistical modeling to predict missing values) was applied.

## Results

Table 1 displays the summary statistics of the multinational population across the TFMI quintiles. This investigation included 27,244 men and 30,520 women. TFMI levels were higher for women than those in men in each quintile; however, BMI was lower among women in quintiles 1 to 4 and only higher in quintile 5. Younger age groups exhibited lower TFMI than the older population. The higher TFMI quintiles were associated with a greater prevalence of diabetes, a trend that was consistent across the UK, US, and South Korean populations. In the multinational population, the prevalence of diabetes among men in TFMI quintile 5 was more than twice than that among those in quintile 1. Additionally, for women, the prevalence in TFMI quintile 5 was 6 times higher than that in quintile 1.

**Table 1:** Descriptive Statistics Across TFMI Quintiles

TFMI quintiles	Men					Women				
	Quintile 1	Quintile 2	Quintile 3	Quintile 4	Quintile 5	Quintile 1	Quintile 2	Quintile 3	Quintile 4	Quintile 5
No. of individuals	5424	5424	5424	5424	5425	6056	6056	6056	6056	6056
TFMI	2.05 (0.54)	3.29 (0.27)	4.19 (0.26)	5.18 (0.33)	7.30 (1.48)	2.46 (0.56)	3.74 (0.29)	4.70 (0.28)	5.84 (0.39)	8.31 (1.65)
Age	60.57 (10.77)	61.18 (10.50)	62.43 (10.01)	63.41 (9.31)	64.05 (8.66)	60.37 (10.45)	60.43 (10.06)	61.74 (9.65)	62.32 (9.41)	61.33 (9.04)
BMI	22.00 (2.09)	24.31 (1.82)	25.79 (2.03)	27.56 (2.09)	31.89 (4.04)	20.76 (1.78)	22.99 (1.61)	24.81 (1.76)	27.21 (2.15)	32.97 (4.86)
Smoking										
Never	2304 (17.49)	2448 (18.59)	2801 (21.27)	2912 (22.11)	2706 (20.55)	4427 (20.53)	4540 (21.05)	4479 (20.77)	4254 (19.72)	3868 (17.93)
Past	1769 (17.26)	2029 (19.79)	2019 (19.70)	2084 (20.33)	2350 (22.92)	1259 (17.98)	1192 (17.02)	1289 (18.41)	1456 (20.79)	1806 (25.79)
Current	1316 (37.49)	906 (25.81)	574 (16.35)	387 (11.03)	327 (9.32)	305 (21.20)	274 (19.04)	245 (17.03)	284 (19.74)	331 (23.00)
Prevalence of diabetes										
Multinational population	374 (6.90)	468 (8.63)	515 (9.49)	521 (9.61)	874 (16.11)	134 (2.22)	260 (4.30)	385 (6.36)	488 (8.06)	823 (13.59)
UK population	97 (2.53)	146 (3.81)	251 (6.55)	312 (8.15)	589 (15.39)	41 (1.01)	50 (1.23)	93 (2.29)	165 (4.06)	383 (9.42)
US population	38 (7.63)	64 (12.85)	103 (20.68)	94 (18.88)	175 (35.14)	22 (4.23)	57 (10.96)	67 (12.88)	121 (23.27)	187 (35.89)
South Korea population	107 (9.75)	143 (13.04)	170 (15.51)	208 (18.94)	255 (23.20)	72 (4.93)	121 (8.27)	166 (11.32)	224 (15.29)	321 (21.85)

Categorical variables: numbers (percentages); continuous variables: mean (SD).

BMI, body mass index; TFMI, Trunk fat mass index.

The RRs of diabetes increased from TFMI quintile 1 to quintile 5 in the unadjusted and adjusted statistical models (Table 2). In the unadjusted model, individuals in TFMI quintile 5 had 2.93 times greater risk of diabetes than those in quintile 1 (95% CI, 2.68-3.22; *P*<.001 for the linear trend). In the model, adjusted for age, sex, ethnicity, smoking status, and BMI, individuals in quintile 5 exhibited 3.38 times higher risk of diabetes than those in quintile 1 (95% CI, 2.95-3.86; *P*<.001 for the linear trend).

**Table 2:** Unadjusted and Adjusted RRs of Diabetes Across Quintiles of TFMI in the Multinational Population

TFMI quintile (mean)	Unadjusted			Linear trend	Adjusted			Linear trend
	RR	95% CI	*P*		RR	95% CI	*P*	
Quintile 1 (2.25)	—	—	—		—	—	—	
Quintile 2 (3.51)	1.30	1.17-1.45	<.001	<.001	1.35	1.21-1.50	<.001	<.001
Quintile 3 (4.45)	1.52	1.37-1.69	<.001		1.70	1.53-1.90	<.001	
Quintile 4 (5.52)	1.81	1.63-1.99	<.001		2.13	1.90-2.38	<.001	
Quintile 5 (7.88)	2.93	2.68-3.22	<.001		3.38	2.95-3.86	<.001	

Covariates, age, gender, ethnicity (UK White, UK mixed, Asian or Asian British, Black or Black British, UK Chinese, UK other ethnic groups, US Mexican American, US other Hispanic, US non-Hispanic White, US non-Hispanic Black, and US other race—including multiracial, and Korean), smoking status, and body mass index.

RR, relative risk; TFMI, trunk fat mass index.

Table 3 portrays the adjusted RRs of diabetes across the TFMI quintiles in BMI-classified groups for men and women. Among men within the normal weight and obese BMI categories, the RRs of diabetes were significantly higher in TFMI quintiles 2 to 5 than those in quintile 1 (*P*≤.04). Overweight men in TFMI quintiles 4 and 5 had significantly higher RRs of diabetes than those in quintile 1 (*P*<.001). Moreover, the RRs of diabetes increased from TFMI quintile 1 to quintile 5, with *P*<.001 for the linear trend among normal weight, overweight, and obese men. For women classified as normal weight and obese using BMI, the RRs of diabetes in TFMI quintiles 3 to 5 were significantly higher than those in quintile 1 (*P*≤.009). The RRs of diabetes among overweight women in TFMI quintiles 4 and 5 were significantly higher than those in quintile 1 (*P*<.001). Furthermore, the RRs of diabetes increased from TFMI quintile 1 to quintile 5 with the linear trend (*P*<.001) among normal weight, overweight, and obese women. For men and women in the normal BMI group, the risk of diabetes was approximately 3 times higher in TFMI quintile 5 than that in quintile 1.

**Table 3:** Adjusted RRs of Diabetes Across Quintiles of TFMI Within Various BMI Categories

TFMI quintile and mean TFMI by sex	Men				Women			
	RR	95% CI	*P*	Linear trend	RR	95% CI	*P*	Linear trend
Normal BMI: mean, 22.629 (M), 22.129 (W)								
TFMI quintile 1; 1.50 (M), 2.09 (W)	1	—	—	<.001	1	—	—	<.001
TFMI quintile 2; 2.31 (M), 2.91 (W)	1.32	1.02-1.72	.04		1.35	0.92-1.98	.13	
TFMI quintile 3; 2.93 (M), 3.48 (W)	1.51	1.13-2.01	.005		1.70	1.14-2.54	.009	
TFMI quintile 4; 3.53 (M), 4.05 (W)	2.18	1.59-2.98	<.001		2.34	1.54-3.56	<.001	
TFMI quintile 5; 4.39 (M), 4.93 (W)	3.06	2.17-4.34	<.001		3.35	2.08-5.39	<.001	
Overweight BMI: mean, 26.738 (M), 26.514 (W)								
TFMI quintile 1; 2.93 (M), 3.94 (W)	1	—	—	<.001	1	—	—	<.001
TFMI quintile 2; 3.91 (M), 4.81 (W)	1.15	0.98-1.34	.09		1.04	0.84-1.28	.73	
TFMI quintile 3; 4.60 (M), 5.40 (W)	1.19	0.98-1.45	.08		1.20	0.96-1.50	.11	
TFMI quintile 4; 5.24 (M), 6.00 (W)	1.56	1.26-1.94	<.001		1.60	1.27-2.01	<.001	
TFMI quintile 5; 6.22 (M), 6.98 (W)	1.92	1.50-2.47	<.001		2.25	1.73-2.91	<.001	
Obese BMI: mean, 33.149 (M), 34.055 (W)								
TFMI quintile 1; 4.63 (M), 6.28 (W)	1	—	—	<.001	1	—	—	<.001
TFMI quintile 2; 6.09 (M), 7.37 (W)	1.29	1.04-1.60	.02		1.24	0.97-1.58	.08	
TFMI quintile 3; 6.95 (M), 8.13 (W)	1.49	1.16-1.91	.002		1.82	1.45-2.29	<.001	
TFMI quintile 4; 7.85 (M), 9.07 (W)	1.79	1.38-2.32	<.001		1.89	1.47-2.44	<.001	
TFMI quintile 5; 9.95 (M), 11.32 (W)	2.47	1.83-3.35	<.001		2.79	2.04-3.83	<.001	

Covariates: age, ethnicity (UK White, UK mixed, Asian or Asian British, Black or Black British, UK Chinese, UK other ethnic groups, US Mexican American, US other Hispanic, US non-Hispanic White, US non-Hispanic Black, and US other race—including multiracial, and Korean), and smoking status.

BMI, body mass index; M, male; RR, relative risk; TFMI, trunk fat mass index; W, women.

The adjusted RRs of diabetes across quintiles of TFMI were estimated for men and women in the UK, US, and South Korea cohorts (Table 4). For both sexes, the RRs of diabetes significantly increased from TFMI quintile 1 to quintile 5, with *P*<.001 for the linear trend in the populations of the United Kingdom, United States, and South Korea. When comparing the RRs in the highest TFMI quintile across the nations, US women exhibited the greatest RR (RR, 10.39; 95% CI, 5.81-18.56), whereas the lowest RR was seen among South Korean men (RR, 1.83; 95% CI, 1.35-2.48). The mean TFMI in each quintile was the highest in the case of US women and lowest for Korean men. The line graphs in Supplemental Figure 1 (available online at http://www.mcpiqojournal.org) showed that individuals with diabetes had higher mean TFMI than those without diabetes (*P*<.001) across all BMI groups.

**Table 4:** Adjusted RRs of Diabetes Across Quintiles of TFMI in the United Kingdom, United States, and South Korea

	Men				Women			
	RR	95% CI	*P*	Linear trend	RR	95% CI	*P*	Linear trend
United Kingdom								
TFMI quintile 1; 2.38 (M), 2.42 (W)	1				1			
TFMI quintile 2; 3.75 (M), 3.78 (W)	1.26	0.98-1.63	.08	<.001	0.99	0.65-1.49	.94	<.001
TFMI quintile 3; 4.65 (M), 4.84 (W)	1.89	1.49-2.40	<.001		1.52	1.04-2.21	.03	
TFMI quintile 4; 5.61 (M), 6.09 (W)	2.04	1.60-2.60	<.001		2.17	1.51-3.11	<.001	
TFMI quintile 5; 7.70 (M), 8.61 (W)	2.89	2.20-3.78	<.001		3.11	2.09-4.63	<.001	
United States								
TFMI quintile 1; 2.20 (M), 2.94 (W)	1							
TFMI quintile 2; 3.33 (M), 4.44 (W)	1.53	1.04-2.26	.03	<.001	2.54	1.56-4.15	<.001	<.001
TFMI quintile 3; 4.08 (M), 5.64 (W)	2.23	1.54-3.22	<.001		3.19	1.93-5.28	<.001	
TFMI quintile 4; 4.89 (M), 6.89 (W)	2.01	1.37-2.96	<.001		6.25	3.75-10.41	<.001	
TFMI quintile 5; 6.93 (M), 9.50 (W)	3.01	1.96-4.60	<.001		10.39	5.81-18.56	<.001	
South Korea								
TFMI quintile 1; 1.51 (M), 2.43 (W)	1							
TFMI quintile 2; 2.38 (M), 3.51 (W)	1.26	0.98-1.61	.07	<.001	1.53	1.15-2.04	.004	<.001
TFMI quintile 3; 2.99 (M), 4.24 (W)	1.43	1.11-1.84	.006		1.84	1.39-2.44	<.001	
TFMI quintile 4; 3.57 (M), 4.97 (W)	1.64	1.26-2.14	<.001		2.12	1.59-2.82	<.001	
TFMI quintile 5; 4.62 (M), 6.37 (W)	1.83	1.35-2.48	<.001		2.59	1.87-3.60	<.001	

Covariates: age, ethnicity (UK White, UK mixed, Asian or Asian British, Black or Black British, UK Chinese, UK other ethnic groups, US Mexican American, US other Hispanic, US non-Hispanic White, US non-Hispanic Black, and US other race—including multiracial, and Korean), and smoking status.

BMI, body mass index; M, male; RR, relative risk; TFMI, trunk fat mass index; W, women.

### Sensitivity Analysis

The adjusted RRs from the modified Poisson regression and adjusted ORs from the logistic regression were compared across the TFMI quintiles. The coefficients for the 2 models were similar, although the ORs from the logistic regression were slightly higher (Supplemental Table 2, available online at http://www.mcpiqojournal.org). The RRs across the TFMI quintiles when using the listwise deletion method vs multiple imputation were estimated, and we found that the results from the 2 methods were comparable (Supplemental Figure 2, available online at http://www.mcpiqojournal.org).

## Discussion

This analysis exhibited a positive association between diabetes and TFMI. Moreover, individuals with higher levels of TFMI have a greater risk of diabetes than those with lower levels, regardless of whether they are classified as normal weight, overweight, or obese based on BMI. Previous studies have found an association between central fat and diabetes; however, there has been a lack of large population-level research investigating diabetes risk linked to varying levels of DXA-measured abdominal fat within specific BMI groups.

A cross-sectional study in the United Kingdom involving 4950 individuals used DXA to measure abdominal fat and found that conventional anthropometry, such as waist circumference, underestimated the association of central fat with T2DM.^30^ Another investigation that was conducted in India and included 1080 participants reported that excess upper body fat assessed using DXA is related to a higher risk of T2DM.^31^ Additionally, a longitudinal study using DXA scans of 30,252 participants from Canada indicated that the risk of diabetes rises with the increase in abdominal fat.^32^

According to the World Health Organization, approximately 830 million people globally have diabetes. The BMI is commonly used worldwide to classify obesity and predict the risk of diabetes. However, BMI is not a direct measure of body fat. Individuals in the normal BMI category but with higher levels of abdominal fat are often misclassified as having a healthy weight. Moreover, those categorized as overweight or obese based on the BMI scale but with low levels of total body or central fat are falsely labeled as being at high risk for diabetes. Research has shown that individuals with a normal BMI but a high BF% or central obesity are at an increased risk for insulin resistance and T2DM.^22^^,^^23^^,^^33^ A study involving 6- to 18-year-old children revealed that greater lean mass had a protective impact on insulin sensitivity among those with high BMI.^34^ It is important to recognize that men and women categorized as normal weight using BMI are not always metabolically healthy; simultaneously, those classified as overweight or obese grade 1 are not necessarily metabolically unhealthy.^35^, ^36^, ^37^, ^38^, ^39^, ^40^, ^41^, ^42^

Advances and technological changes in DXA devices have enhanced the ability to assess BF, fat-free mass and bone mass density using a 3-compartment model.^43^ Body fat assessments conducted by GE-Lunar iDXA and Hologic QDR 4500A fan-beam densitometers are known to have high accuracy, reproducibility and reliability, making the measurements by these 2 devices highly comparable. Dual-energy X-ray absorptiometry is certainly safe, with an exposure level lower than a daily dose of natural background radiation. Computed tomography and magnetic resonance imaging are considered superior in differentiating adipose tissue (intramuscular, subcutaneous, and visceral); however, they are expensive to maintain. Furthermore, a single exposure to a computed tomography scan is equivalent to years of background radiation.^44^^,^^45^ Ultrasound sonography can also differentiate fat tissue; however, its reproducibility with BC assessment is lower than the other imaging techniques owing to a lack of standardized scanning protocols.^46^ Considering the strengths and weaknesses of these imaging techniques, DXA emerges as the best option for assessing BC. Routine BC examinations in a primary care setting can aid in diagnosing unhealthy BC and predicting diabetes risk. Furthermore, data generated from the DXA assessments may contribute to addressing important clinical research questions.

BC is considered unhealthy when there are higher or lower than the optimal levels of BF%^1^^,^^2^ or high-fat deposits around the abdomen.^47^^,^^48^ The American College of Sports Medicine has classified healthy BF% levels for men and women across different age groups in the Guidelines for Exercise Testing and Prescription.^49^ However, there are no established classification or cutoff values for intra-abdominal fat or visceral adipose tissue, and it may constitute 10% to 20% of total BF in men and 5% to 8% among women, which tends to increase with age.^47^^,^^50^^,^^51^ BC is a byproduct of an individual’s lifestyle (amount and intensity of physical activity and dietary patterns),^52^^,^^53^ and it also reflects one’s level of cardiorespiratory fitness.^54^, ^55^, ^56^, ^57^ Determining a person’s physical activity and diet can be complex; however, BC parameters can be assessed directly using DXA or other imaging techniques.^52^^,^^53^^,^^57^^,^^58^

### Strengths and Limitations

The data for this analysis consisted of individuals with DXA-measured TFM from 3 different regions of the world. Dual-energy X-ray absorptiometry scanning is considered the gold standard for assessing body fat because of its high accuracy.^43^ To our knowledge, this analysis is novel because it used the largest sample size ever examined to study the association between DXA-measured central fat and diabetes. Participants from the United States and South Korea were representative of their general population, whereas those from the United Kingdom were relatively healthy, with evidence of sampling bias.^59^^,^^60^ Only the data from the United Kingdom were linked to primary care and hospital records concerning diabetes history; therefore, the prevalence was estimated via a self-reported diabetes history and blood glucose levels in the harmonized central data set to ensure homogeneity.

Thorax consists of visceral and subcutaneous fat, whereas limb fat largely comprises subcutaneous fat.^61^ The analysis did not include limb fat mass, which is known to improve insulin sensitivity, and future research should demonstrate its role in chronic metabolic disorders.^62^ Moreover, data on physical activity, including aerobic or resistance exercise,^41^^,^^57^^,^^63^^,^^64^ cardiorespiratory fitness, or muscular strength,^65^, ^66^, ^67^, ^68^ were not pooled, which are known important predictors of diabetes risk or prognosis and obesity or unhealthy BC. Self-reported diabetes status was not differentiated as T2DM and type 1 diabetes mellitus in the data sets from the United Kingdom, United States, and South Korea. Therefore, the reported prevalence of diabetes includes individuals who self-reported having T2DM or type 1 diabetes mellitus, those taking hypoglycemic medications or those with elevated HbA1c or blood glucose levels. Because this is an observational study, the associations between TFMI and diabetes status should not be interpreted to imply causality.

## Conclusion

High levels of TFMI are associated with an increased risk of diabetes among men and women. Additionally, individuals with higher central fat are more likely to experience diabetes than those with lower TFM, even if they are classified within the same BMI groups, including normal weight, overweight, or obese. Directly measured abdominal fat using imaging techniques could facilitate better prediction of diabetes risk and prognosis. Future research should aim to establish unhealthy threshold levels for TFMI or central fat.

## Potential Competing Interests

Given his role as Editorial Board Member, Dr Lavie was not involved in the peer review of this article and has no access to information regarding its peer review. The other authors report no competing interests.

## Ethics Statement

This study was approved by North West Multi-centre Research Ethics Committee (UKB study), National Center for Health Statistics Research Ethics Review Board (US NHANES), Institutional Review Board of the Korea Disease Control and Prevention Agency (KNHANES). Consent documents were signed by all the participants in the UKB study, US NHANES, and KNHANES. C.O., M.K., C.J.L., H.O., M.-J.S., X.S., J.J., R.P., J.G., and V.K.P take responsibility for the integrity of the data used in the analysis and are the guarantors of this study.

## References

[1] Wells J.C.K., Shirley M.K. (2016). Body composition and the monitoring of non-communicable chronic disease risk. Glob Health Epidemiol Genom.

[2] Wells J.C.K. (2019). Body composition of children with moderate and severe undernutrition and after treatment: a narrative review. BMC Med.

[3] Bergman R.N., Kim S.P., Catalano K.J. (2006). Why visceral fat is bad: mechanisms of the metabolic syndrome. Obesity (Silver Spring).

[4] Cruz-Jentoft A.J., Sayer A.A. (2019). Sarcopenia. Lancet.

[5] Frankenfield D.C., Rowe W.A., Cooney R.N., Smith J.S., Becker D. (2001). Limits of body mass index to detect obesity and predict body composition. Nutrition.

[6] DeFronzo R.A., Gunnarsson R., Björkman O., Olsson M., Wahren J. (1985). Effects of insulin on peripheral and splanchnic glucose metabolism in noninsulin-dependent (type II) diabetes mellitus. J Clin Invest.

[7] Chadt A., Al-Hasani H. (2020). Glucose transporters in adipose tissue, liver, and skeletal muscle in metabolic health and disease. Pflugers Arch.

[8] Ito H., Nakasuga K., Ohshima A. (2003). Detection of cardiovascular risk factors by indices of obesity obtained from anthropometry and dual-energy x-ray absorptiometry in Japanese individuals. Int J Obes Relat Metab Disord.

[9] Papaetis G.S., Papakyriakou P., Panagiotou T.N. (2015). Central obesity, type 2 diabetes and insulin: exploring a pathway full of thorns. Arch Med Sci.

[10] Trouwborst I., Bowser S.M., Goossens G.H., Blaak E.E. (2018). Ectopic fat accumulation in distinct insulin resistant phenotypes; targets for personalized nutritional interventions. Front Nutr.

[11] Gravina G., Ferrari F., Nebbiai G. (2021). The obesity paradox and diabetes. Eat Weight Disord.

[12] Shulman G.I. (2014). Ectopic fat in insulin resistance, dyslipidemia, and cardiometabolic disease. N Engl J Med.

[13] Narayan K.M., Boyle J.P., Thompson T.J., Gregg E.W., Williamson D.F. (2007). Effect of BMI on lifetime risk for diabetes in the U.S. Diabetes Care.

[14] Arnlöv J., Sundström J., Ingelsson E., Lind L. (2011). Impact of BMI and the metabolic syndrome on the risk of diabetes in middle-aged men. Diabetes Care.

[15] Tirosh A., Shai I., Afek A. (2011). Adolescent BMI trajectory and risk of diabetes versus coronary disease. N Engl J Med.

[16] Mokdad A.H., Ford E.S., Bowman B.A. (2003). Prevalence of obesity, diabetes, and obesity-related health risk factors, 2001. JAMA.

[17] Teufel F., Seiglie J.A., Geldsetzer P. (2021). Body-mass index and diabetes risk in 57 low-income and middle-income countries: a cross-sectional study of nationally representative, individual-level data in 685 616 adults. Lancet.

[18] Logue J., Walker J.J., Leese G. (2013). Association between BMI measured within a year after diagnosis of type 2 diabetes and mortality. Diabetes Care.

[19] Lin C.C., Li C.I., Liu C.S. (2019). Obesity paradox in associations between body mass index and diabetes-related hospitalization and mortality in patients with type 2 diabetes: retrospective cohort studies. Diabetes Metab.

[20] Lajous M., Bijon A., Fagherazzi G. (2014). Body mass index, diabetes, and mortality in French women: explaining away a “paradox”. Epidemiology.

[21] Kwon Y., Kim H.J., Park S., Park Y.G., Cho K.H. (2017). Body mass index-related mortality in patients with type 2 diabetes and heterogeneity in obesity paradox studies: a dose-response meta-analysis. PLoS One.

[22] Sánchez-Castillo C.P., Velásquez-Monroy O., Lara-Esqueda A. (2005). Diabetes and hypertension increases in a society with abdominal obesity: results of the Mexican National Health Survey 2000. Public Health Nutr.

[23] Kapoor N., Feingold K.R., Ahmed S.F., Anawalt B. (2000). EndoText.

[24] UK Biobank About Our Data. https://www.ukbiobank.ac.uk/about-our-data/.

[25] Centers for Disease Control and Prevention (2020).

[26] Kweon S., Kim Y., Jang M.J. (2014). Data resource profile: the Korea National Health and Nutrition Examination Survey (KNHANES). Int J Epidemiol.

[27] Kim Y. (2014). The Korea National Health and Nutrition Examination Survey (KNHANES): current status and challenges. Epidemiol Health.

[28] Fortier I., Raina P., Van den Heuvel E.R. (2017). Maelstrom Research guidelines for rigorous retrospective data harmonization. Int J Epidemiol.

[29] Wey T.W., Doiron D., Wissa R. (2021). Overview of retrospective data harmonisation in the MINDMAP project: process and results. J Epidemiol Community Health.

[30] Vasan S.K., Osmond C., Canoy D. (2018). Comparison of regional fat measurements by dual-energy x-ray absorptiometry and conventional anthropometry and their association with markers of diabetes and cardiovascular disease risk. Int J Obes (Lond).

[31] Gowri S.M., Antonisamy B., Geethanjali F.S. (2021). Distinct opposing associations of upper and lower body fat depots with metabolic and cardiovascular disease risk markers. Int J Obes (Lond).

[32] Leslie W.D., Ludwig S.M., Morin S. (2010). Abdominal fat from spine dual-energy x-ray absorptiometry and risk for subsequent diabetes. J Clin Endocrinol Metab.

[33] Oliveros E., Somers V.K., Sochor O., Goel K., Lopez-Jimenez F. (2014). The concept of normal weight obesity. Prog Cardiovasc Dis.

[34] Xiao P., Cheng H., Yan Y. (2021). High BMI with adequate lean mass is not associated with cardiometabolic risk factors in children and adolescents. J Nutr.

[35] Wei M., Kampert J.B., Barlow C.E. (1999). Relationship between low cardiorespiratory fitness and mortality in normal-weight, overweight, and obese men. JAMA.

[36] Nogueira E.C., Porto L.G., Nogueira R.M. (2016). Body composition is strongly associated with cardiorespiratory fitness in a large Brazilian Military Firefighter cohort: the Brazilian Firefighters study. J Strength Cond Res.

[37] Barry V.W., Baruth M., Beets M.W., Durstine J.L., Liu J., Blair S.N. (2014). Fitness vs. fatness on all-cause mortality: a meta-analysis. Prog Cardiovasc Dis.

[38] Church T.S., LaMonte M.J., Barlow C.E., Blair S.N. (2005). Cardiorespiratory fitness and body mass index as predictors of cardiovascular disease mortality among men with diabetes. Arch Intern Med.

[39] Blair S.N., Kohl H.W., Barlow C.E., Paffenbarger R.S., Gibbons L.W., Macera C.A. (1995). Changes in physical fitness and all-cause mortality. A prospective study of healthy and unhealthy men. JAMA.

[40] Pandey A., Patel K.V., Vaduganathan M. (2018). Physical activity, fitness, and obesity in heart failure with preserved ejection fraction. JACC Heart Fail.

[41] Lavie C.J., Ozemek C., Carbone S., Katzmarzyk P.T., Blair S.N. (2019). Sedentary behavior, exercise, and cardiovascular health. Circ Res.

[42] Lavie C.J., Laddu D., Arena R., Ortega F.B., Alpert M.A., Kushner R.F. (2018). Healthy weight and obesity prevention: JACC Health Promotion Series. J Am Coll Cardiol.

[43] Bazzocchi A., Ponti F., Albisinni U., Battista G., Guglielmi G. (2016). DXA: technical aspects and application. Eur J Radiol.

[44] Erlandson M.C., Lorbergs A.L., Mathur S., Cheung A.M. (2016). Muscle analysis using pQCT, DXA and MRI. Eur J Radiol.

[45] Guglielmi G., Ponti F., Agostini M., Amadori M., Battista G., Bazzocchi A. (2016). The role of DXA in sarcopenia. Aging Clin Exp Res.

[46] Wagner D.R. (2013). Ultrasound as a tool to assess body fat. J Obes.

[47] Ibrahim M.M. (2010). Subcutaneous and visceral adipose tissue: structural and functional differences. Obes Rev.

[48] Tchernof A., Després J.P. (2013). Pathophysiology of human visceral obesity: an update. Physiol Rev.

[49] Riebe D., Ehrman J.K., Liguori G., Magal M., American College of Sports Medicine (2018).

[50] Huffman D.M., Barzilai N. (2009). Role of visceral adipose tissue in aging. Biochim Biophys Acta.

[51] Wajchenberg B.L. (2000). Subcutaneous and visceral adipose tissue: their relation to the metabolic syndrome. Endocr Rev.

[52] Nishikori S., Fujita S. (2024). Association of fat-to-muscle mass ratio with physical activity and dietary protein, carbohydrate, sodium, and fiber intake in a cross-sectional study. Sci Rep.

[53] Padua E., Caprio M., Feraco A. (2023). The impact of diet and physical activity on fat-to-lean mass ratio. Nutrients.

[54] Blair S.N., Kampert J.B., Kohl H.W. (1996). Influences of cardiorespiratory fitness and other precursors on cardiovascular disease and all-cause mortality in men and women. JAMA.

[55] Sui X., LaMonte M.J., Laditka J.N. (2007). Cardiorespiratory fitness and adiposity as mortality predictors in older adults. JAMA.

[56] Lavie C.J., Sanchis-Gomar F., Ozemek C. (2022). Fit is it for longevity across populations. J Am Coll Cardiol.

[57] Liu Y., Lee D.C., Li Y. (2019). Associations of resistance exercise with cardiovascular disease morbidity and mortality. Med Sci Sports Exerc.

[58] Willoughby D., Hewlings S., Kalman D. (2018). Body composition changes in weight loss: strategies and supplementation for maintaining lean body mass, a brief review. Nutrients.

[59] Fry A., Littlejohns T.J., Sudlow C. (2017). Comparison of sociodemographic and health-related characteristics of UK Biobank participants with those of the general population. Am J Epidemiol.

[60] Batty G.D., Gale C.R., Kivimäki M., Deary I.J., Bell S. (2020). Comparison of risk factor associations in UK Biobank against representative, general population based studies with conventional response rates: prospective cohort study and individual participant meta-analysis. BMJ.

[61] Montague C.T., O’Rahilly S. (2000). The perils of portliness: causes and consequences of visceral adiposity. Diabetes.

[62] Jung S., Park J., Seo Y.G. (2021). Relationship between arm-to-leg and limbs-to-trunk body composition ratio and cardiovascular disease risk factors. Sci Rep.

[63] Cabanas-Sánchez V., Duarte Junior M.A., Lavie C.J., Celis-Morales C., Rodríguez-Artalejo F., Martínez-Gómez D. (2024). Physical activity and cause-specific cardiovascular mortality among people with and without cardiovascular disease: a cohort study of 0.6 million US adults. Mayo Clin Proc.

[64] Martinez-Gomez D., Rodriguez-Artalejo F., Ding D., Ekelund U., Cabanas-Sanchez V. (2024). Trends in the association between meeting the physical activity guidelines and risk of mortality in US adults. Prog Cardiovasc Dis.

[65] Carbone S., Kirkman D.L., Garten R.S. (2020). Muscular strength and cardiovascular disease: an updated state-of-the-art narrative review. J Cardiopulm Rehabil Prev.

[66] Lavie C.J., Laukkanen J.A., Nath K.A. (2025). Cardiorespiratory fitness and Mayo Clinic Proceedings. Mayo Clin Proc.

[67] Ross R., Arena R., Myers J., Kokkinos P., Kaminsky L.A. (2024). Update to the 2016 American Heart Association cardiorespiratory fitness statement. Prog Cardiovasc Dis.

[68] Laukkanen J.A., Isiozor N.M., Kunutsor S.K. (2022). Objectively assessed cardiorespiratory fitness and all-cause mortality risk: an updated meta-analysis of 37 cohort studies involving 2,258,029 participants. Mayo Clin Proc.

