# Supplementary material for: Association Between Trunk Fat Mass Index and Diabetes in a Multinational Population
Source: Mayo Clin Proc Innov Qual Outcomes. 2025 Sep 19;9(5):100658. doi: 10.1016/j.mayocpiqo.2025.100658 (PMC12482302; doi:10.1016/j.mayocpiqo.2025.100658)

Supplementary Material

Supplementary Table 1. Data harmonisation

**Variable name**	**Variable description and harmonisation**		
	South Korea	UK	USA
Sex	Self-reported via an in-person interview	Acquired from the central registry of the National Health Services and updated by the participant during the interview	Self-reported via an in-person interview or telephone interview
Age	Self-reported via an in-person interview	Acquired from the central registry of the National Health Services and updated by the participant during the interview	Self-reported via an in-person interview or telephone interview
Ethnicity	Generated variable	Self-Reported via questionnaire	Self-reported through an in-person or telephone interview
Trunk fat mass	Analysed via a Dual-energy X-ray absorptiometry (QDR 4500A fan-beam densitometer) during the years 2008-2011	Analysed via a Dual-energy X-ray absorptiometry (Hologic, Inc. GE-Lunar, Madison, WI, USA fan-beam densitometer) during the year 2014-2023	Analysed via a Dual-energy X-ray absorptiometry (QDR 4500A fan-beam densitometer) during the years 2011 to 2018
Body mass index	Height and weight were measured during physical examination. BMI was calculated using height and weight	Height and weight were measured during physical examination. BMI was calculated using height and weight	Height and weight were measured during physical examination. BMI was calculated using height and weight
Smoking-status	Smoking status was self-reported. Based on self-report, individuals were classified as:<br>1. Current smokers; smoked ≥ 100 cigarettes during their lifetime and were currently smoking either every day or some days<br>2. Former smokers; smoked ≥ 100 cigarettes during their lifetime but currently do not smoke<br>3. Never smoked; reported never having smoked 100 cigarettes during their lifetime	Smoking status was self-reported. Based on self-report, individuals were classified as:<br>1. Current smokers; smoked ≥ 100 cigarettes during their lifetime and were currently smoking either every day or some days<br>2. Former smokers; smoked ≥ 100 cigarettes during their lifetime but currently do not smoke<br>3. Never smoked; reported never having smoked 100 cigarettes during their lifetime	Smoking status was self-reported. Based on self-report, individuals were classified as:<br>1. Current smokers; smoked ≥ 100 cigarettes during their lifetime and were currently smoking either every day or some days<br>2. Former smokers; smoked ≥ 100 cigarettes during their lifetime but currently do not smoke<br>3. Never smoked; reported never having smoked 100 cigarettes during their lifetime

Diabetes and blood glucose investigation	1. Diabetes status was self-reported via an in-person interview<br>2. Currently on insulin treatment and/or use of oral hypoglycemic drugs<br>3. Diabetes status was directly assessed: fasting blood sugar≥126mg/dl confirmed diabetes	1. Diabetes status was self-reported via an in-person interview<br>2. Currently on insulin treatment and/or use of oral hypoglycemic drugs<br>3. Diabetes status was directly assessed: HbA1c ≥ 48 mmol/mol (6.5%) confirmed diabetes	1. Diabetes status was self-reported via an in-person interview<br>2. Currently on insulin treatment and/or use of oral hypoglycemic drugs<br>3. Diabetes status was directly assessed: HbA1c ≥ 48 mmol/mol (6.5%) confirmed diabetes

Supplementary Table 2: Relative risks of diabetes from the modified Poisson regression model versus odds ratios from the logistic regression model (Adjusted models)

	Modified Poisson regression				Logistic regression			
	RR	95% CI	P-value	Linear Trend	OR	95% CI	P-value	Linear Trend
TFMI Quintile 2	1.35	(1.21, 1.50)	<0.001	<0.001	1.37	(1.21, 1.55)	<0.001	<0.001
TFMI Quintile 3	1.70	(1.53, 1.90)	<0.001		1.76	(1.55,1.99)	<0.001	
TFMI Quintile 4	2.13	(1.90, 2.38)	<0.001		2.23	(1.95, 2.54)	<0.001	
TFMI Quintile 5	3.38	(2.95, 3.86)	<0.001		3.70	(3.13, 4.37)	<0.001	

TFMI: Trunk fat mass index, RR: Relative risk, OR: Odds ratio, CI: Confidence interval
Covariates: Age, gender, ethnicity (UK white, UK mixed, Asian or Asian British, Black or Black British, UK Chinese, UK other ethnic groups, US Mexican American, US other Hispanic, US Non-Hispanic White, US Non-Hispanic Black, US other race - including multi-racial, and Korean), smoking status and body mass index.

Supplementary Figure 1. Mean trunk fat mass index (TFMI) among individuals with diabetes versus those without diabetes across Body mass index (BMI) categories

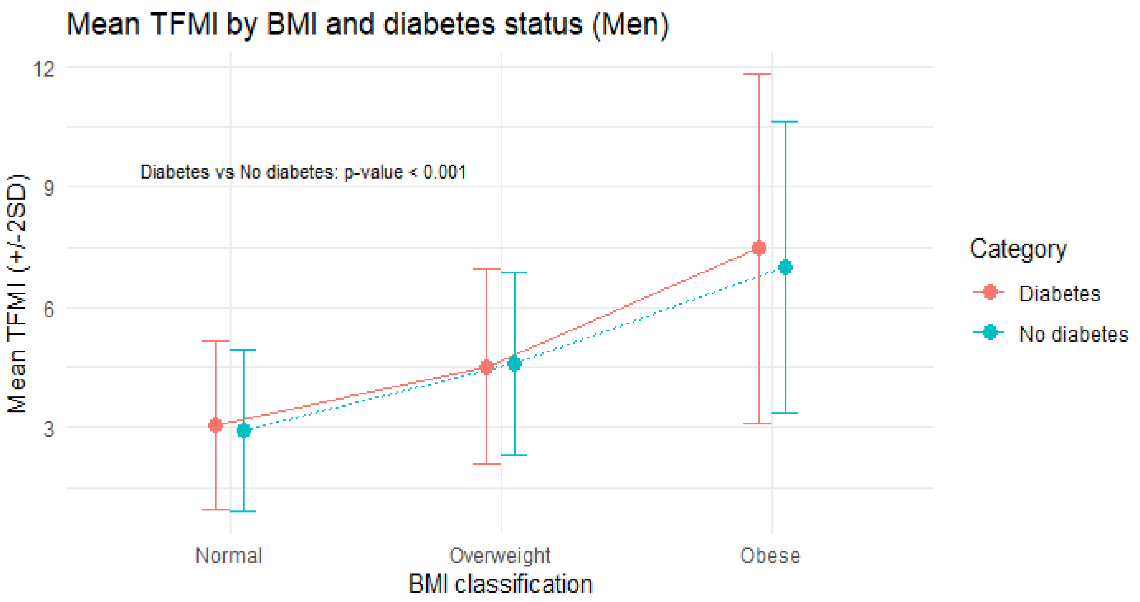

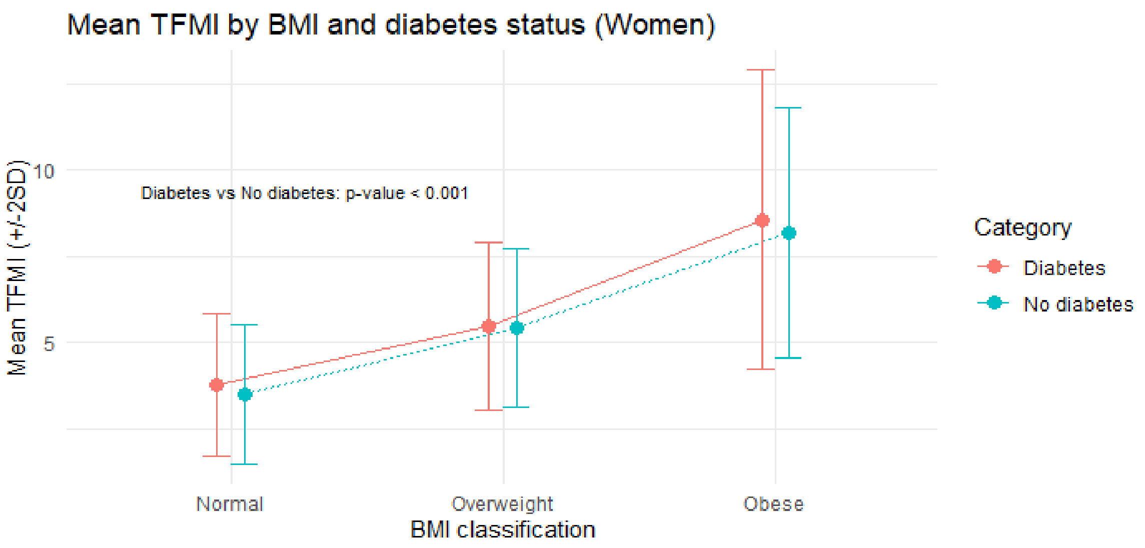

Supplementary Figure 2: Adjusted relative risks (RRs) of diabetes across trunk fat mass index (TFMI) quintiles, comparing results when missing values are handled by listwise deletion versus multiple imputation.

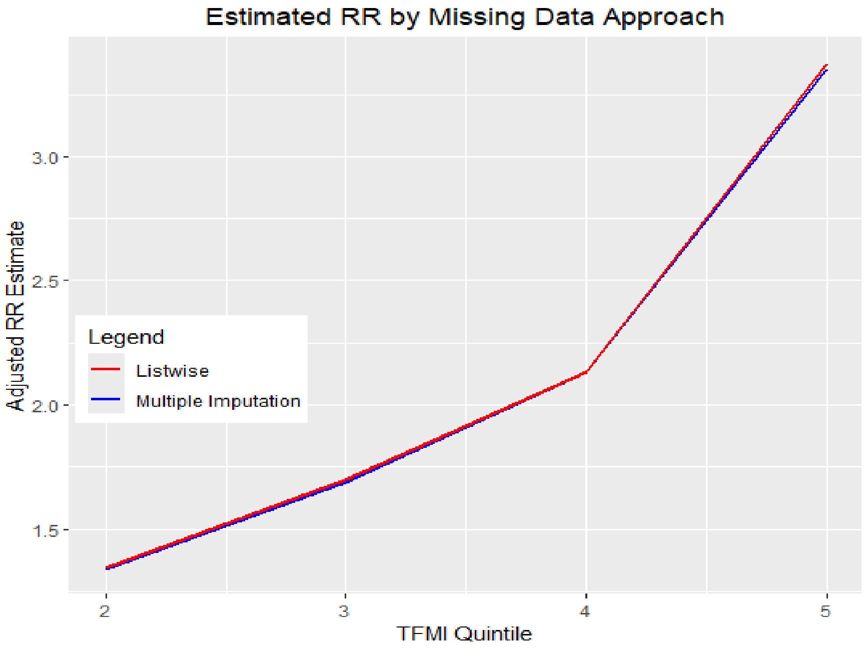

Supplement: Supplementary Tables 1 and 2 and Supplementary Figures 1 and 2 [file mmc1.pdf]
